# Comparative Bioaccumulation of Mineral Elements in Soft Tissues, Shells, and Pearls of *Sinohyriopsis cumingii* (Lea, 1852) Cultured for Non-Nucleated, Visceral Mass-Nucleated, and Mantle-Nucleated Pearls

**DOI:** 10.3390/ani16203231

**Published:** 2026-10-14

**Authors:** Ibrahim Bah, Mengying Gu, Meiyi Wang, Haibo Li, Haibo Wen, Junren Xue, Xiubao Chen

**Affiliations:** 1 Wuxi Fisheries College, Nanjing Agricultural University, Wuxi 214081, China; 2 Ministry of Fisheries and Marine Resources, Freetown 190, Sierra Leone; 3 College of Marine Science and Technology and Environment, Dalian Ocean University, Dalian 116023, China; 4 Jilin Provincial Academy of Fishery Sciences, Changchun 130033, China; 5 Freshwater Fisheries Research Center, Chinese Academy of Fishery Sciences, Wuxi 214081, China

**Keywords:** freshwater pearl mussel, minerals, pearl quality, ICP-MS

## Abstract

The triangle sail mussel *Sinohyriopsis cumingii* (Lea, 1852) is the world’s leading producer of freshwater pearls. Mineral elements are key components of pearls and affect pearl quality, but their bioaccumulation patterns in the soft tissues, shells, and pearls of this species remain unclear. Inductively coupled plasma mass spectrometry was used to compare the bioaccumulation of essential major elements (Na, Mg, K, Ca), essential trace elements (Cr, Mn, Fe, Co, Ni, Cu, Zn, Se, Mo), and non-essential elements (Al, As, Sr, Ag, Cd, Ba, Tl, Pb) in the soft tissues, shells, and pearls of *S. cumingii* cultured for non-nucleated, visceral mass-nucleated, and mantle-nucleated pearls. Mg, K, Mn, Zn, and Ba were significantly more concentrated in soft tissues than in shells or pearls, whereas Ca and Sr showed the opposite trend (*p* < 0.05). Principal component analysis based on Zn, Ca, Mg, Sr, and Mn separated soft tissues from shells and pearls, while a second component based on Mo, Fe, Co, and Ni distinguished non-nucleated from visceral mass-nucleated and mantle-nucleated pearl mussels. Ca–Sr and Sr–Ba were consistently positively correlated in soft tissues across all three culture types (*p* < 0.05). Notably, Na was frequently involved in negative correlations between tissues: in mussels cultured for non-nucleated pearls, shell Na was negatively correlated with soft tissue K and pearl Na with soft tissue Ba (*p* < 0.05); in those cultured for visceral mass-nucleated pearls, soft tissue Na was negatively correlated with pearl Mn and Zn (*p* < 0.05); and in those cultured for mantle-nucleated pearls, pearl Na was negatively correlated with shell Zn (*p* < 0.05). Collectively, these findings elucidate mineral element bioaccumulation in pearl-producing *S. cumingii*.

## Simple Summary

Freshwater pearls are a valuable product, with China being the world’s largest producer. Farmers use different methods to grow pearls in a mussel species called *Sinohyriopsis cumingii* (Lea, 1852). These methods can produce many small pearls or fewer, larger, rounder ones. However, how the mussels take up minerals from the water to build their shells and pearls remains poorly understood. This study measured 21 different minerals in the soft body, shell, and pearls of mussels grown using three different pearl-culture methods. We found that mineral levels differed among the three culture groups, although the relative contributions of culture method, mussel age, and culture duration could not be distinguished. Minerals like magnesium and manganese were mostly stored in the mussel’s soft body, while calcium and strontium were mainly found in the shell and pearl. Notably, sodium in one tissue was often negatively correlated with other elements in another tissue. For instance, higher sodium levels in the soft body were linked to lower manganese and zinc in the pearls, two elements that affect pearl color. This research provides a basis for future hypothesis-driven studies aimed at testing whether targeted elemental regulation can influence freshwater pearl quality, such as color.

## 1. Introduction

The triangle sail mussel *Sinohyriopsis cumingii* (Lea, 1852) originated in China [1] and is now also distributed in Japan and Vietnam [2,3]. It is an important freshwater pearl-producing mussel [1,2,3,4]. In China, the world’s largest producer of freshwater pearls today, *S. cumingii* produces the vast majority of pearls [3,4,5,6]. The traditional method of pearl production using *S. cumingii* primarily involves implanting small pieces of mantle tissue obtained from a donor mussel into the mantle of a host mussel to culture non-nucleated pearls. Typically, each host mussel can produce approximately 30 non-nucleated pearls. Although this method yields a high output, the long cultivation period (3–5 years) and low proportion of high-quality pearls (roundness rate < 5%) result in relatively low economic value.

To address the challenge of “high output but low value” associated with non-nucleated pearls, visceral mass-nucleated pearl production was introduced around 2011 [7,8]. This technique involves implanting a single large nucleus (typically 5–8 mm in diameter), together with a small piece of mantle tissue obtained from a donor mussel, into the visceral mass of a host mussel [7,8]. Compared with non-nucleated pearls, visceral mass-nucleated pearl production offers several advantages, including a significantly shorter cultivation period (1–2 years) and the production of larger, more spherical pearls, with roundness rates of up to 40%. However, this method also presents notable limitations, such as low production output (only one pearl per mussel) and a relatively high rejection rate of the implanted nucleus (approximately 20%).

In response to these limitations, mantle-nucleated pearl production has emerged as a new cultivation approach in recent years [9]. This method involves implanting multiple small nuclei (generally 10–30 nuclei, each 1–2 mm in diameter), together with small pieces of mantle tissue obtained from a donor mussel, into the mantle of a host mussel. This approach not only shortens the cultivation period but also improves both pearl quality and yield. Consequently, it has gained considerable popularity in commercial cultivation.

During pearl cultivation, *S. cumingii* absorbs and accumulates various mineral elements, including both essential and non-essential elements, from the aquatic environment, which subsequently affects mussel growth and development as well as pearl quality [10]. For example, when the Fe concentration in the substrate ranges from 1.75% to 4.75%, it promotes the growth of soft tissues and shells of *S. cumingii*, whereas exceeding this range inhibits growth [6]. The accumulation of Co, Ti, Ag, Mn, Mg, Zn, Fe, and/or Cu in pearls plays an important role in determining pearl color in *S. cumingii* [11,12,13]. It should be noted that the bioaccumulation of mineral elements in pearl mussels is not independent but rather exhibits correlations. For instance, Ca accumulation in the soft tissues of *S. cumingii* inhibits Cd absorption [14]. However, the accumulation characteristics of mineral elements in the soft tissues, shells, and pearls of pearl mussels remain unclear.

This study aims to compare the concentrations of mineral elements, including essential major elements (Na, Mg, K, Ca), essential trace elements (Cr, Mn, Fe, Co, Ni, Cu, Zn, Se, Mo), and non-essential elements (Al, As, Sr, Ag, Cd, Ba, Tl, Pb), in the soft tissues, shells, and pearls of *S. cumingii* cultured for non-nucleated pearls, visceral mass-nucleated pearls, and mantle-nucleated pearls, and subsequently analyze the principal components and correlation patterns of mineral elements within and between different tissues. We hypothesize that the bioaccumulation of mineral elements in the soft tissues, shells, and pearls of *S. cumingii* exhibits specificity and coupling. The results will elucidate the bioaccumulation characteristics of mineral elements in pearl-producing *S. cumingii* and provide a basis for future hypothesis-driven studies aimed at testing whether targeted elemental regulation can influence freshwater pearl quality.

## 2. Materials and Methods

### 2.1. Mussel Collection

**Figure 1:**
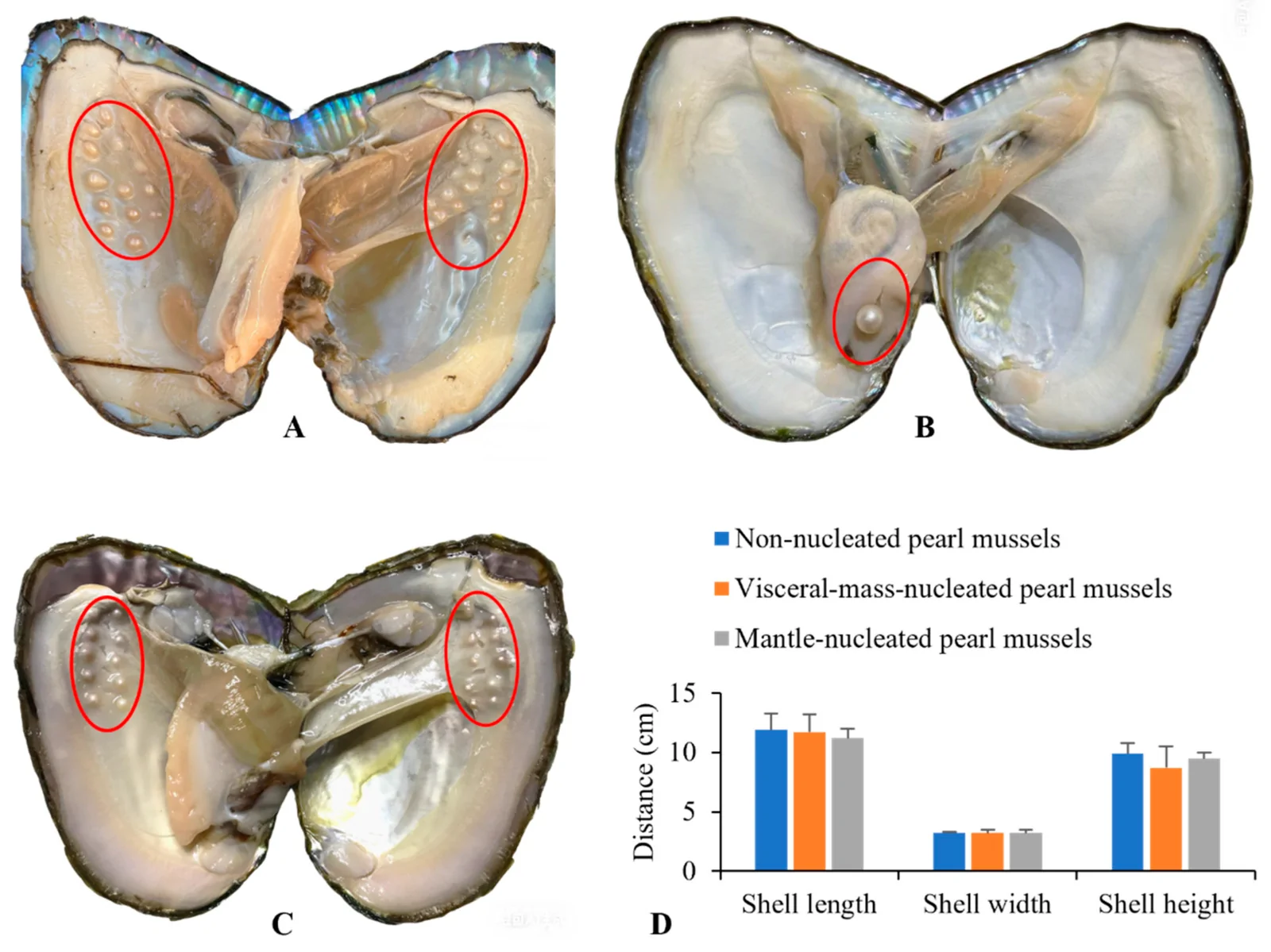
*Sinohyriopsis cumingii* specimens cultured for non-nucleated pearls (**A**), visceral mass-nucleated pearls (**B**), and mantle-nucleated pearls (**C**), along with their sizes (**D**). Red circles indicate the pearl growth sites.

The *S. cumingii* mussels cultured for non-nucleated pearls (Figure 1A, *n* = 7), visceral mass-nucleated pearls (Figure 1B, *n* = 10), and mantle-nucleated pearls (Figure 1C, *n* = 10) were all collected from the Nanquan Aquaculture Base of the Freshwater Fisheries Research Center, Chinese Academy of Fishery Sciences (Wuxi, China). The three pearl-culture groups originated from a single cohort. The Nanquan Aquaculture Base is a closed, pollution-free scientific research station [15]. The concentration ranges of Al, Cr, Mn, Fe, Ni, Cu, Zn, As, Mo, Cd, Ba, Tl, and Pb in its water were 1.53–176, 0–0.75, 0–8.01, 17.41–1094, 0–1.71, 0–0.88, 1.4–97, 1.8–3.55, 1.17–2.09, 0–0.27, 32.22–65.4, 0–1.23, and 0–1.18 µg/L, respectively [15]. Previous studies have demonstrated that 7–10 samples are sufficient to adequately characterize the mineral element accumulation patterns of pearl mussels or pearls [16,17]. The mussels cultured for non-nucleated pearls were 3 years old, with a pearl culture period of 2 years; whereas the mussels cultured for visceral mass-nucleated pearls and mantle-nucleated pearls were both 2 years old, with a pearl culture period of 1 year. The longer culture period for non-nucleated pearls was due to their slower formation rate. Nevertheless, the mussel sizes were comparable (Figure 1D). After collection, attached materials on the shells were removed, and the mussels were subsequently transferred to aerated tap water for a 72 h depuration period to empty their intestinal contents.

### 2.2. Processing of Soft Tissues, Shells, and Pearls

The entire soft tissues, whole shells, and all pearls were dissected from the mussels using a stainless steel scalpel. The soft tissues and pearls were cleaned with ultrapure water (Millipore Corp., Burlington, VT, USA, with a resistivity of 18.2 MΩ·cm) [18,19]. To remove potential residual materials from the shell surfaces, the shells were thoroughly cleaned following the method described by Cravo et al. [20]. Briefly, the shells were first soaked overnight at 60 °C in 50% (*w*/*v*) H_2_O_2_ at a ratio of 1 mL/g shell, followed by overnight soaking in 0.05 M HCl at a ratio of 1 mL/g shell. The shells were then thoroughly rinsed with ultrapure water.

After cleaning, all samples were dried at 80 °C for 24 h until a constant weight was reached. Soft tissues were subsequently ground into a homogeneous powder using an agate mortar. Due to their large size and hardness, shell samples were crushed and homogenized using a mill (IKA A11, Staufen, Germany). Non-nucleated pearls were mechanically fragmented and ground into a homogeneous powder using an agate mortar. For visceral mass-nucleated pearls and mantle-nucleated pearls, the pearls were carefully broken to remove the nuclei, after which the nacre was ground into a homogeneous powder using an agate mortar.

### 2.3. Mineral Element Analysis

Following our previous descriptions [18,21], the concentrations of essential major elements (Na, Mg, K, Ca), essential trace elements (Cr, Mn, Fe, Co, Ni, Cu, Zn, Se, Mo), and non-essential elements (Al, As, Sr, Ag, Cd, Ba, Tl, Pb) were determined in soft tissue, shell, and pearl samples. Briefly, each dry sample (0.1 ± 0.005 g) was placed into a Teflon digestion tube and 5 mL of HNO_3_ (Merck, Darmstadt, Germany, 65%) was added. All mixtures were digested for 10 min at 120 °C, 15 min at 170 °C, and a further 15 min at 170 °C using an ETHOS A T260 microwave digestion system (Milestone Inc., Sorisole, Italy). The obtained solutions were diluted to 100 mL with ultra-pure water. Element concentrations were analyzed using an Agilent 7500ce Inductive Coupled Plasma—Mass Spectrometry (ICP-MS; Agilent Technologies, USA). Matrix–effects and instrumental drift of the ICP–MS were corrected by Li, Sc, Ge, Y, In, and Bi as the internal standard. Analytical quality was checked using a commercial standard solution (Agilent Technologies, Santa Clara, CA, USA) and the certified reference material DOLT-5 dogfish liver (National Research Council of Canada, Ottawa, ON, Canada). Element recoveries ranged from 90% to 110%. The instrument sensitivity is lower than 0.001 µg/g for almost all the elements investigated. Values below the detection limit were replaced with half of the detection limit (0.0005 µg/g) for all statistical analyses.

### 2.4. Statistical Analysis

All statistical analyses were performed using *R* software (version 4.5.2). Given the small sample size (*n* = 7–10), non-parametric tests were used. For comparisons of element concentrations within the same individual, the Friedman test was used, followed by the Nemenyi test. For comparisons of element concentrations among the three pearl-culture groups, the Kruskal–Wallis test was used, followed by Dunn’s test with Holm adjustment. Principal component analysis (PCA) was utilized to assess the comprehensive accumulation profiles of mineral elements in soft tissue, shell, and pearl. The PCA was performed on the original, untransformed data. Spearman correlation analysis with Benjamini–Hochberg FDR correction was used to reveal the associations of mineral element accumulation both within and between different tissues. A *p*-value (adjusted where applicable) of less than 0.05 was considered statistically significant.

## 3. Results

### 3.1. Mineral Elements in the Soft Tissues, Shells, and Pearls of Mussels Cultured for Non-Nucleated Pearls

#### 3.1.1. Mineral Element Concentrations

The concentrations of mineral elements in the soft tissues are shown in Table 1. Among the essential major elements, Ca differed significantly from Mg and K (*p* < 0.05). Among the essential trace elements, Mn and Fe differed significantly from Co, Ni, Cu, and Se, while Zn differed from Co and Se (*p* < 0.05). Among the non-essential elements, Sr and Ba differed significantly from Cd, and Ba differed from As (*p* < 0.05). The concentration order was: Ca > Mn ≈ Fe > Na ≈ Mg ≈ K ≈ Ba > Zn ≈ Sr > Al ≈ Cr ≈ As ≈ Mo > Cu ≈ Ni ≈ Co ≈ Cd.

In both the shells and the pearls (Table 1), among the essential major elements, Ca differed significantly from Mg and K, and Na differed significantly from Mg (*p* < 0.05). Among the essential trace elements, Fe differed significantly from Cu, Zn, Se, and Mo, while Mn and Ni differed significantly from Cu, Zn, and Se (*p* < 0.05). Among the non-essential elements, both Sr and Ba differed significantly from As and Cd (*p* < 0.05). The overall concentration order was: Ca > Fe > Na > Sr > Mn ≈ Ba ≈ K ≈ Mg > Ni ≈ Al ≈ Co ≈ Cr ≈ Mo ≈ Se.

The element concentrations in the soft tissues, shells, and pearls were compared (Table 1). In the soft tissues, the concentrations of Mg, K, Zn, and As were the highest, whereas those of Fe and Co were the lowest (Holm-adjusted *p* < 0.05). Mn and Ba showed similar concentrations in the soft tissues and pearls (Holm-adjusted *p* > 0.05), but were significantly higher than those in the shells (Holm-adjusted *p* < 0.05). In the shells and pearls, Ca and Sr concentrations were similar (Holm-adjusted *p* > 0.05), but were significantly higher than those in the soft tissues (Holm-adjusted *p* < 0.05).

#### 3.1.2. Principal Component Analysis

**Figure 2:**
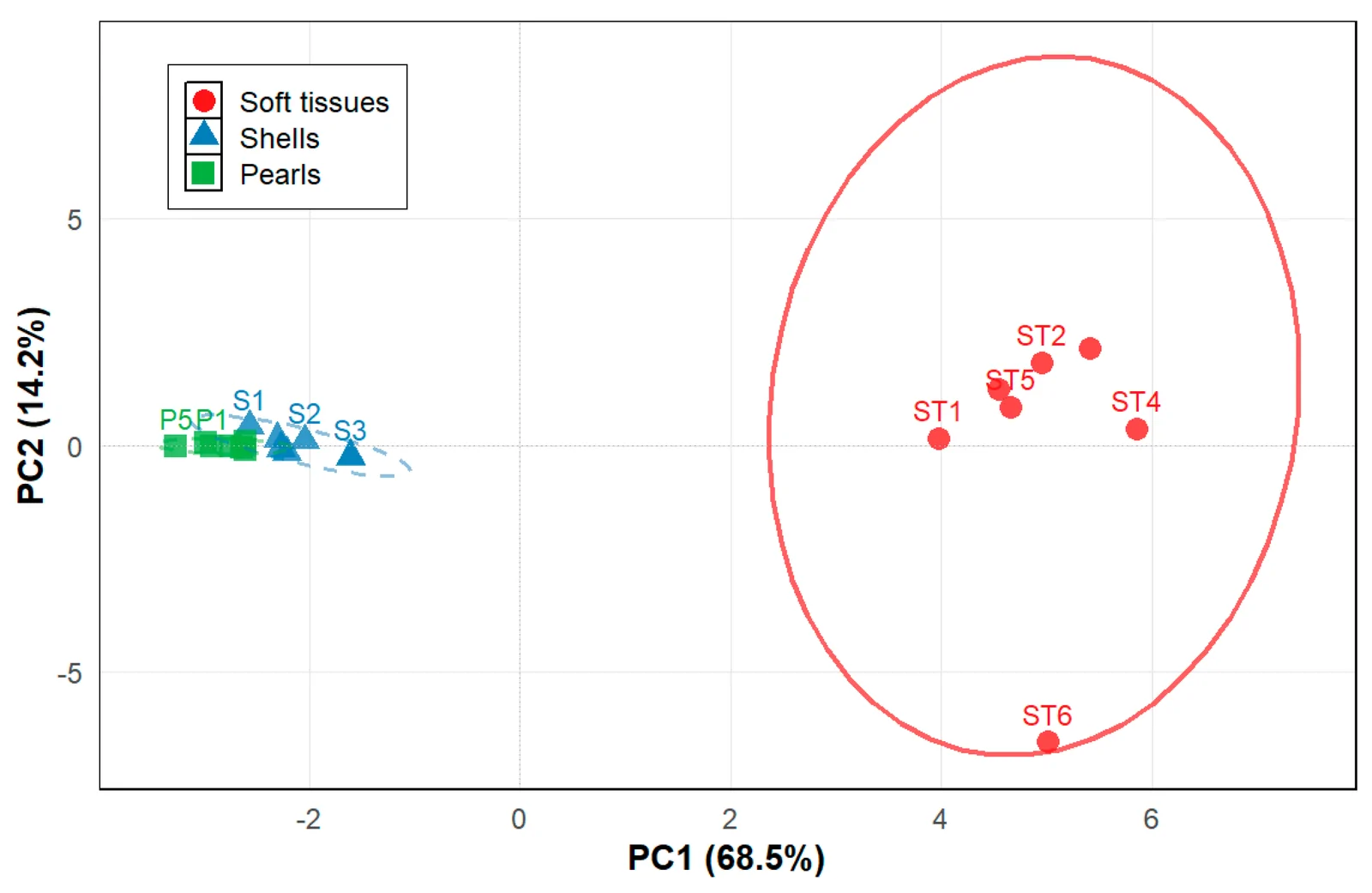
Principal component analysis of mineral elements in the soft tissues, shells, and pearls of *Sinohyriopsis cumingii* cultured for non-nucleated pearls (*n* = 7).

As shown in Figure 2, PC1 and PC2 cumulatively explained 82.7% of the variation in mineral element concentrations among the soft tissues, shells, and pearls. The main contributing elements to PC1 were Ca (7.97%), Fe (7.84%), Zn (7.77%), Co (7.76%), Sr (7.53%), K (7.51%), Mg (7.49%), Mn (7.35%), and As (7.12%). The main contributing elements to PC2 were Cd (32.91%), Na (28.74%), and Cu (22.69%). The centroid values for soft tissues, shells, and pearls on PC1 and PC2 were (4.914, 0.009), (−2.090, −0.002), and (−2.825, −0.006), respectively.

#### 3.1.3. Correlation Analysis

**Figure 3:**
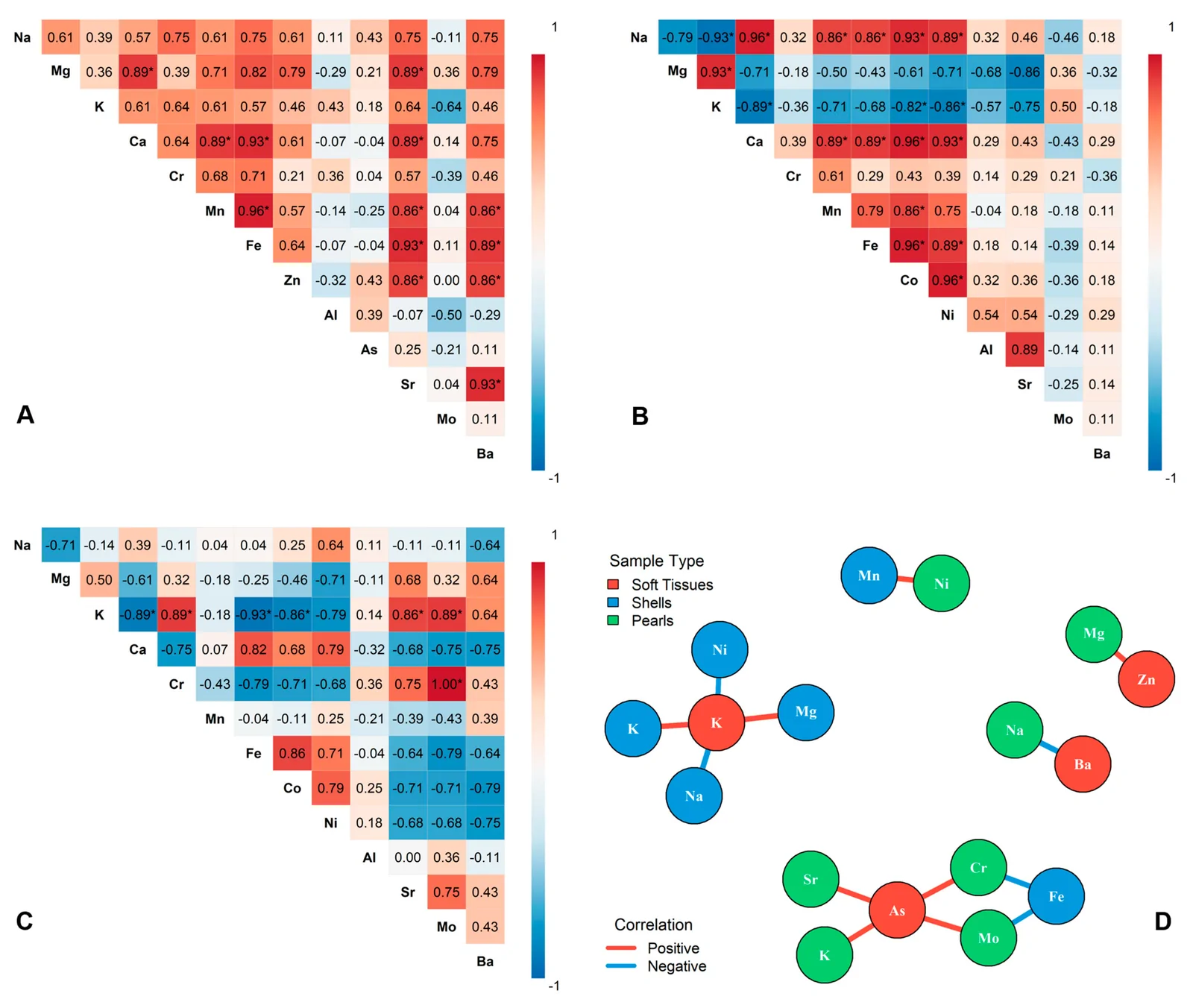
Spearman correlation analysis of mineral elements in the soft tissues (**A**), shells (**B**), pearls (**C**), and among them (**D**) of *Sinohyriopsis cumingii* cultured for non-nucleated pearls (*n* = 7). * indicates a significant correlation after Benjamini–Hochberg FDR correction (adjusted *p* < 0.05).

Correlation patterns of mineral elements varied among the soft tissues, shells, and pearls (Figure 3A–C). In the soft tissues, Mg was positively correlated with Ca and Sr; Ca was positively correlated with Mn, Fe, and Sr; Mn was positively correlated with Fe, Sr, and Ba; Fe and Zn were each positively correlated with Sr and Ba; and Sr was positively correlated with Ba (FDR-adjusted *p* < 0.05). In the shells, Na was positively correlated with Ca, Mn, Fe, Co, and Ni; Mg was positively correlated with K; Ca was positively correlated with Mn, Fe, Co, and Ni; Mn was positively correlated with Co; Fe was positively correlated with Co and Ni; whereas K was negatively correlated with Na, Ca, Co, and Ni (FDR-adjusted *p* < 0.05). In the pearls, K was positively correlated with Cr, Sr, and Mo, but was negatively correlated with Ca, Fe, and Co; and Cr was negatively correlated with Mo (FDR-adjusted *p* < 0.05).

Correlations of mineral elements between the soft tissues, shells, and pearls are shown in Figure 3D. Between the soft tissues and shells, soft tissue K was positively correlated with shell Mg and K, but negatively correlated with shell Na and Ni (FDR-adjusted *p* < 0.05). Between the soft tissues and pearls, Zn was positively correlated with Mg; As was positively correlated with K, Cr, Sr, and Mo; whereas Ba was negatively correlated with Na (FDR-adjusted *p* < 0.05). Between the shells and pearls, Mn was positively correlated with Ni, and Fe was positively correlated with Cr and Mo (FDR-adjusted *p* < 0.05).

### 3.2. Mineral Elements in the Soft Tissues, Shells, and Pearls of Mussels Cultured for Visceral Mass-Nucleated Pearls

#### 3.2.1. Mineral Element Concentrations

The concentrations of mineral elements in the soft tissues are shown in Table 2. Among the essential major elements, Ca differed significantly from Na and Mg, while Mg differed from K (*p* < 0.05). Among the essential trace elements, Mn differed significantly from Zn (*p* < 0.05). Among the non-essential elements, Al differed significantly from Ba, and As differed from Sr (*p* < 0.05). The concentration order was: Ca > Mn ≈ K ≈ Na ≈ Mg > Fe ≈ Ba ≈ Zn > Sr > Al > As.

In the shells (Table 2), among the essential major elements, Ca differed significantly from Mg and K, and Na differed significantly from K (*p* < 0.05). Among the essential trace elements, Mn differed significantly from Cr and Zn (*p* < 0.05). Among the non-essential elements, Sr differed significantly from Al (*p* < 0.05). The concentration order was: Ca > Na > Sr > Mn > Mg ≈ Ba > Zn ≈ Cr ≈ Al.

In the pearls (Table 2), among the essential major elements, Ca differed significantly from Mg and K, and Na differed significantly from K (*p* < 0.05). Among the essential trace elements, Mn differed significantly from Cr (*p* < 0.05). Among the non-essential elements, Sr differed significantly from Al (*p* < 0.05). The concentration order was: Ca > Na > Sr > Mn > Mg > Ba > Zn ≈ Al.

The element concentrations in the soft tissues, shells, and pearls were compared (Table 2). In the soft tissues, the concentrations of Mg, K, Mn, Fe, Zn, Al, As, and Ba were the highest, significantly higher than those in the shells and pearls (Holm-adjusted *p* < 0.05). In contrast, the concentrations of Na, Ca, and Sr in the shells and pearls were significantly higher than those in the soft tissues (Holm-adjusted *p* < 0.05).

#### 3.2.2. Principal Component Analysis

**Figure 4:**
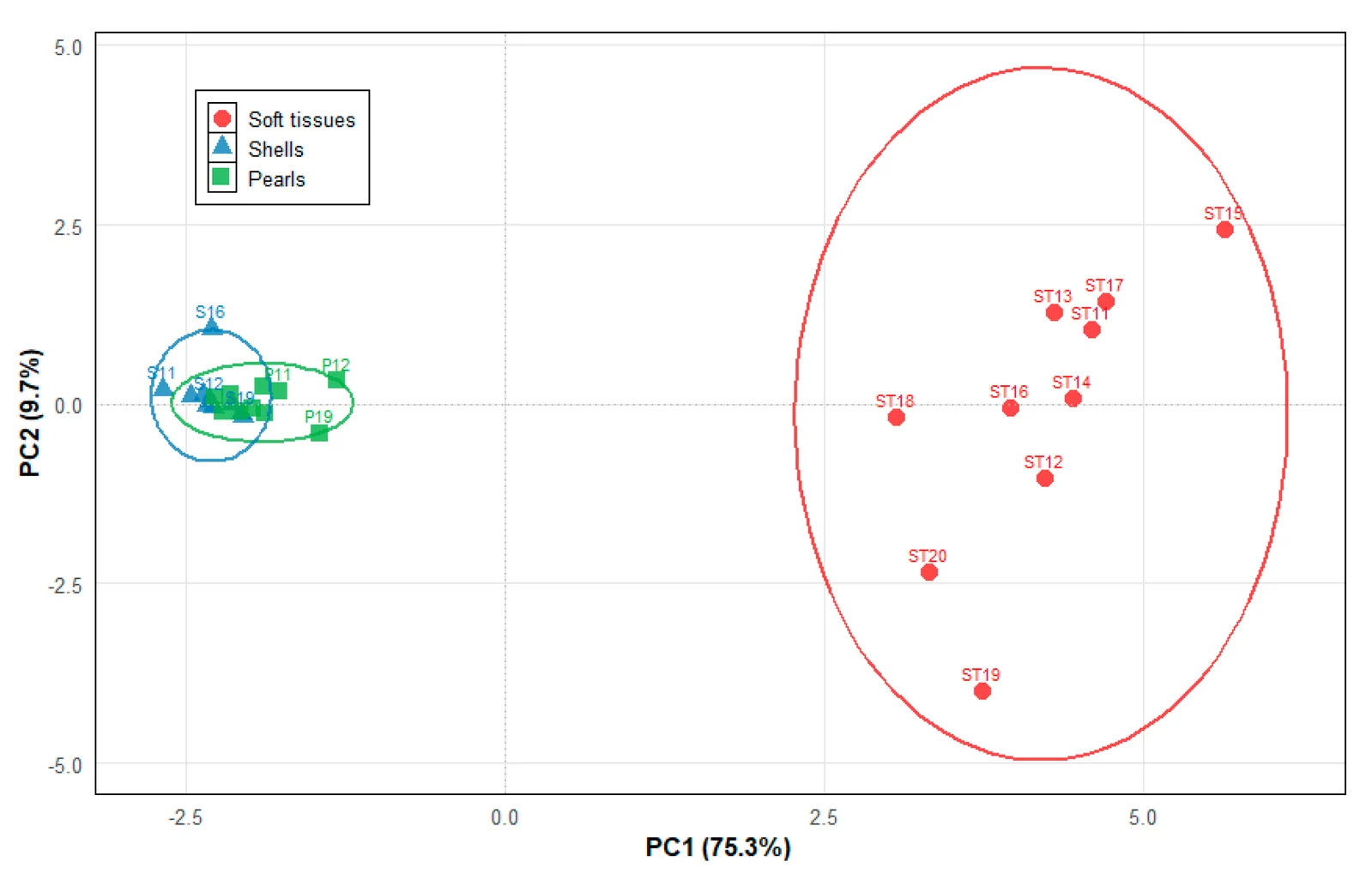
Principal component analysis of mineral elements in the soft tissues, shells, and pearls of *Sinohyriopsis cumingii* cultured for visceral mass-nucleated pearls (*n* = 10).

As shown in Figure 4, PC1 and PC2 cumulatively explained 85.0% of the variation in mineral element concentrations among the soft tissues, shells, and pearls. The main contributing elements to PC1 were Zn (10.98%), Ca (10.78%), Mg (10.76%), Mn (10.72%), K (10.52%), and Fe (10.33%), whereas the main contributing elements to PC2 were As (56.94%) and Al (23.16%). The centroid values for soft tissues, shells, and pearls on PC1 and PC2 were (4.198, −0.135), (−2.299, 0.116), and (−1.899, 0.019), respectively.

#### 3.2.3. Correlation Analysis

**Figure 5:**
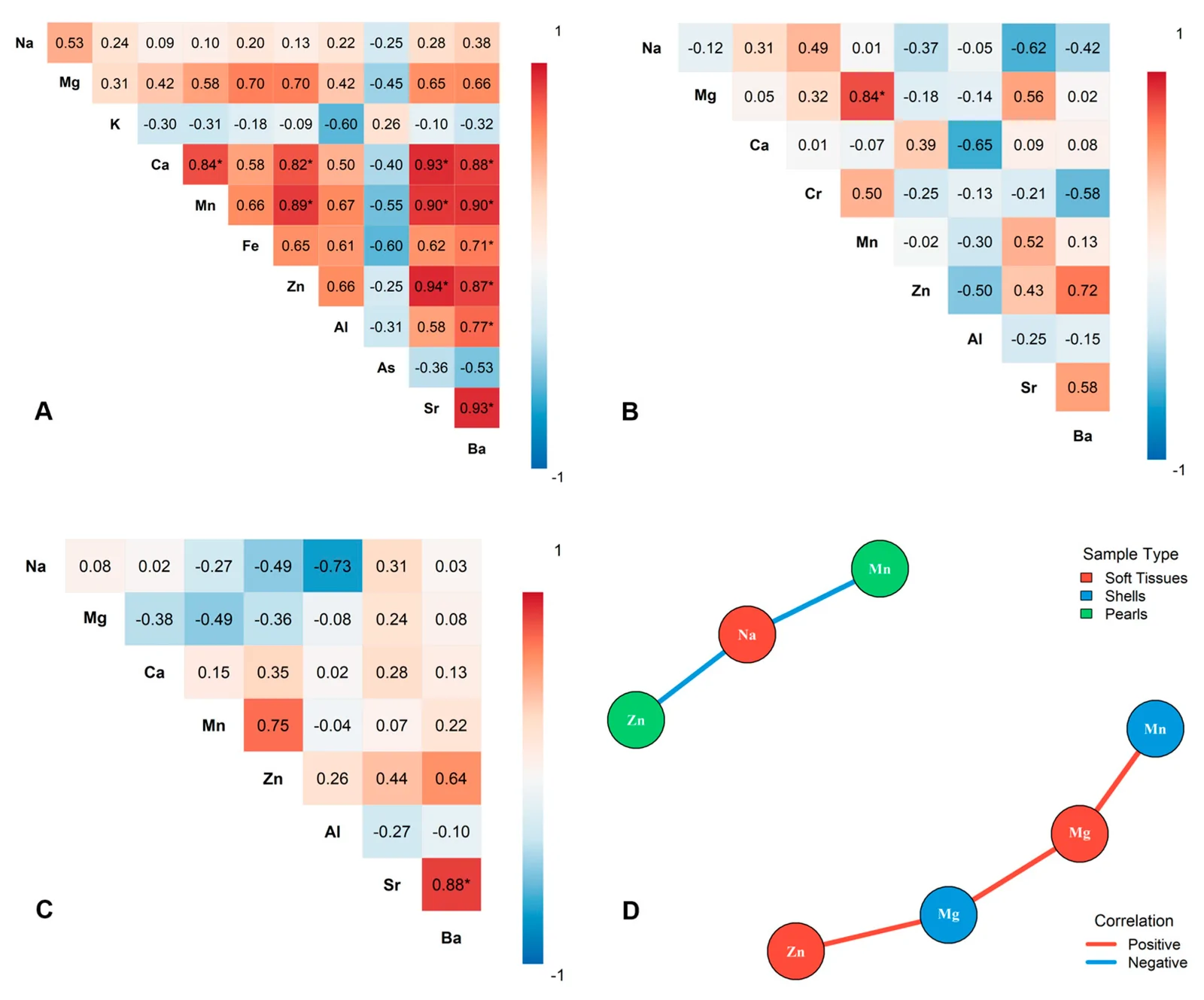
Spearman correlation analysis of mineral elements in the soft tissues (**A**), shells (**B**), pearls (**C**), and among them (**D**) of *Sinohyriopsis cumingii* cultured for visceral mass-nucleated pearls (*n* = 10). * indicates a significant correlation after Benjamini–Hochberg FDR correction (adjusted *p* < 0.05).

The correlations among mineral elements in the soft tissues, shells, and pearls are shown in Figure 5. In the soft tissues, Ca was positively correlated with Mn, Zn, Sr, and Ba; Mn was positively correlated with Zn, Sr, and Ba; Zn was positively correlated with Sr and Ba; and Sr was positively correlated with Ba (FDR-adjusted *p* < 0.05). In the shells, Mg was positively correlated with Mn (FDR-adjusted *p* < 0.05). In the pearls, Sr was positively correlated with Ba (FDR-adjusted *p* < 0.05).

Correlations of mineral elements between the soft tissues, shells, and pearls are shown in Figure 5D. Soft tissue Mg was significantly positively correlated with shell Mg and Mn, and soft tissue Zn was significantly positively correlated with shell Mg (FDR-adjusted *p* < 0.05). In addition, soft tissue Na was significantly negatively correlated with pearl Mn and Zn (FDR-adjusted *p* < 0.05).

### 3.3. Mineral Elements in the Soft Tissues, Shells, and Pearls of Mussels Cultured for Mantle-Nucleated Pearls

#### 3.3.1. Mineral Element Concentrations

The concentrations of mineral elements in the soft tissues are shown in Table 3. Among the essential major elements, Ca differed significantly from Na and Mg, and Mg differed from K (*p* < 0.05). Among the essential trace elements, Mn differed significantly from Fe and Zn (*p* < 0.05). Among the non-essential elements, Ba differed significantly from Al and As, and Sr differed from As (*p* < 0.05). The concentration order was: Ca > Mn ≈ K ≈ Na ≈ Mg > Fe ≈ Ba ≈ Zn > Sr > Al ≈ As.

In the shells (Table 3), among the essential major elements, Ca differed significantly from Mg (*p* < 0.05). Among the essential trace elements, Mn differed significantly from Cr and Se, and Zn differed from Se (*p* < 0.05). Among the non-essential elements, Sr differed significantly from Al (*p* < 0.05). The concentration order was: Ca > Na > Sr > Mn > Mg > Ba > Zn ≈ Al.

In the pearls (Table 3), among the essential major elements, Ca differed significantly from Mg (*p* < 0.05). Among the essential trace elements, Mn differed significantly from Zn (*p* < 0.05). Among the non-essential elements, Sr differed significantly from Al (*p* < 0.05). The overall concentration order was: Ca > Na > Sr > Mn > Mg > Ba > Zn ≈ Al.

The element concentrations in the soft tissues, shells, and pearls were compared (Table 3). In the soft tissues, the concentrations of Mg, K, Mn, Fe, Zn, Al, and Ba were the highest, significantly higher than those in the shells and pearls (Holm-adjusted *p* < 0.05). Conversely, the concentrations of Ca and Sr in the shells and pearls were significantly higher than those in the soft tissues (Holm-adjusted *p* < 0.05). Additionally, the Na concentration in the pearls was significantly higher than those in the soft tissues and shells (Holm-adjusted *p* < 0.05).

#### 3.3.2. Principal Component Analysis

**Figure 6:**
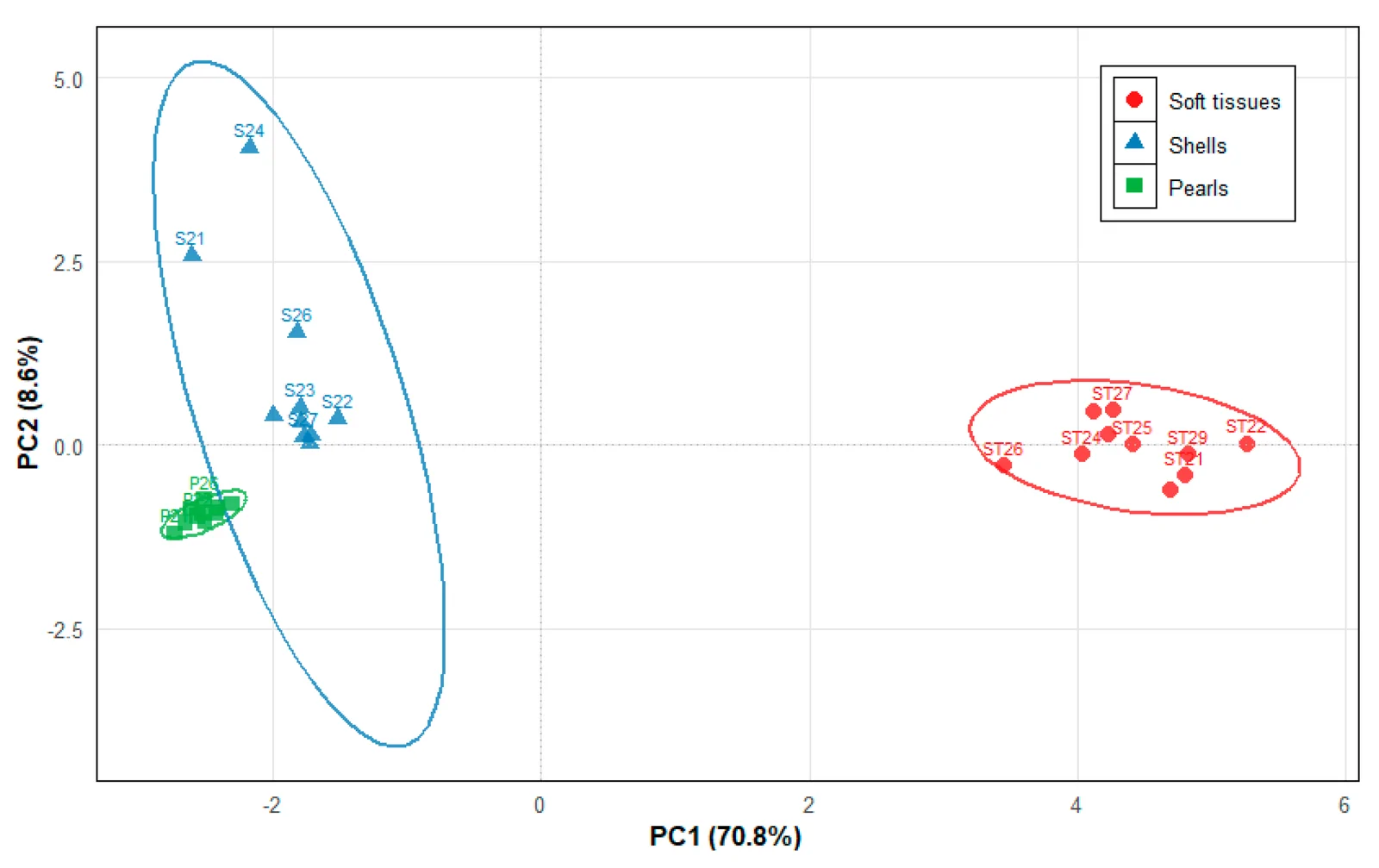
Principal component analysis of mineral elements in the soft tissues, shells, and pearls of *Sinohyriopsis cumingii* cultured for mantle-nucleated pearls (*n* = 10).

As shown in Figure 6, PC1 and PC2 cumulatively explained 79.4% of the variation in mineral element concentrations among the soft tissues, shells, and pearls. The main contributing elements to PC1 were Ca (9.87%), Mg (9.86%), Zn (9.86%), K (9.83%), Mn (9.54%), Fe (9.52%), and Ba (9.42%), whereas the main contributing elements to PC2 were Se (45.97%), Cr (25.43%), and Na (11.15%). The centroid values for soft tissues, shells, and pearls on PC1 and PC2 were (4.41, −0.05), (−1.89, 0.996), and (−2.52, −0.947), respectively.

#### 3.3.3. Correlation Analysis

**Figure 7:**
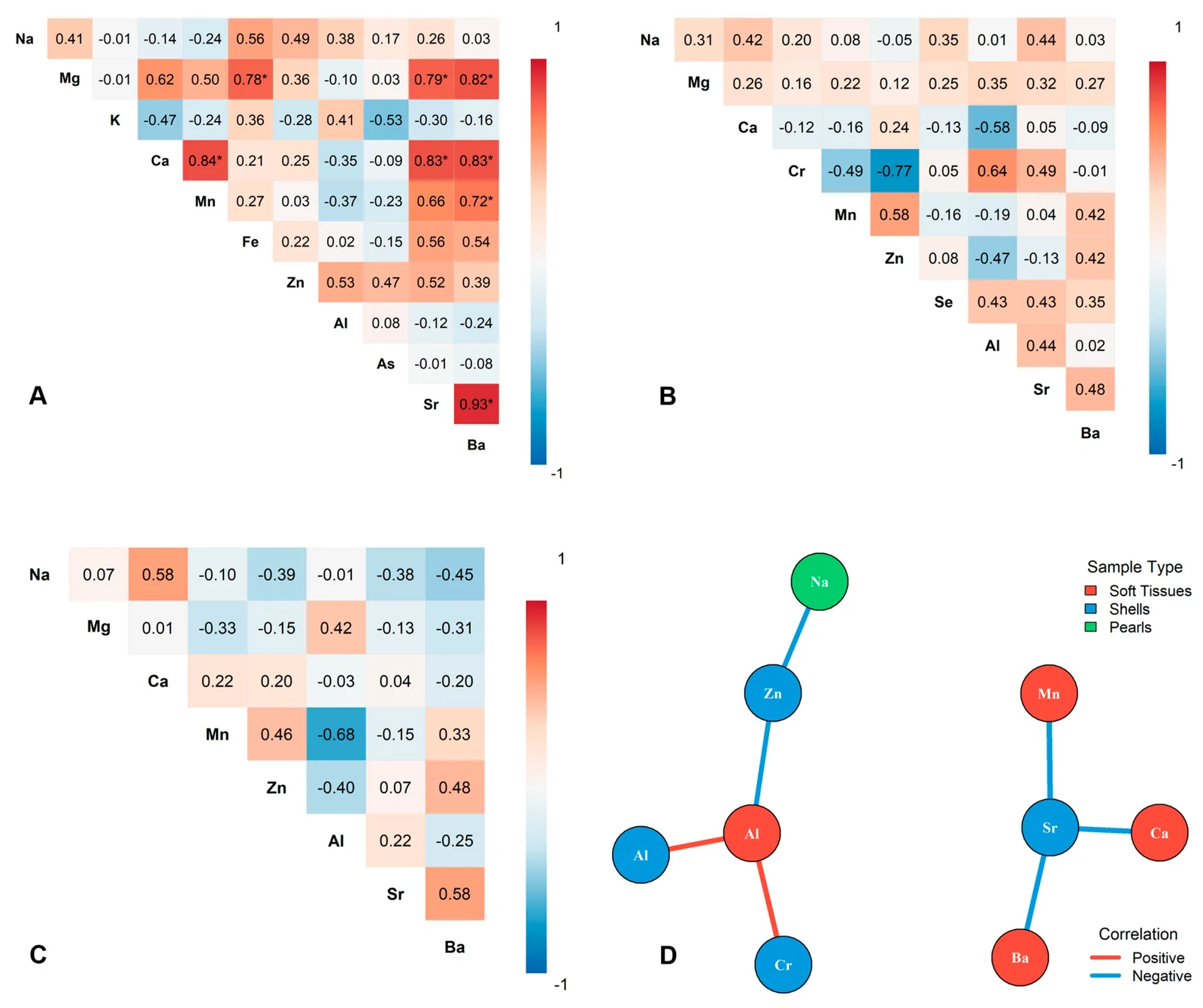
Spearman correlation analysis of mineral elements in the soft tissues (**A**), shells (**B**), pearls (**C**), and among them (**D**) of *Sinohyriopsis cumingii* cultured for mantle-nucleated pearls (*n* = 10). * indicates a significant correlation after Benjamini–Hochberg FDR correction (adjusted *p* < 0.05).

The correlations among mineral elements in the soft tissues, shells, and pearls are shown in Figure 7. In the soft tissues, Mg was positively correlated with Fe, Sr, and Ba; Ca was positively correlated with Mn, Sr, and Ba; and both Mn and Sr were positively correlated with Ba (FDR-adjusted *p* < 0.05). No significant correlations were found among elements in the shells or pearls (FDR-adjusted *p* > 0.05).

Correlations of mineral elements between the soft tissues, shells, and pearls are shown in Figure 7D. Soft tissue Al was positively correlated with shell Al and Cr, but negatively correlated with Zn (FDR-adjusted *p* < 0.05). Soft tissue Ca, Mn, and Ba were each negatively correlated with shell Sr (FDR-adjusted *p* < 0.05). Shell Zn was negatively correlated with pearl Na (FDR-adjusted *p* < 0.05). No significant correlations were found between the soft tissues and pearls (FDR-adjusted *p* > 0.05).

### 3.4. Comparison of Mineral Elements in Soft Tissues, Shells, and Pearls of Mussels Cultured for Different Pearl Types

#### 3.4.1. Mineral Element Concentrations

Mineral elements in the soft tissues were compared among mussels cultured for different pearl types. Ca, Cr, Mn, Fe, As, Sr, Mo, and Ba were significantly higher in mussels cultured for non-nucleated pearls than in those cultured for visceral mass-nucleated and mantle-nucleated pearls (Holm-adjusted *p* < 0.05), while their concentrations were similar between the latter two groups (Holm-adjusted *p* > 0.05). K was similar between mussels cultured for visceral mass-nucleated and mantle-nucleated pearls (Holm-adjusted *p* > 0.05), but significantly higher than in mussels cultured for non-nucleated pearls (Holm-adjusted *p* < 0.05). The remaining elements showed similar concentrations among the three groups (Holm-adjusted *p* > 0.05).

In the shells, Mg, K, Fe, Co, Ni, Al, Mo, and Ba were significantly higher in mussels cultured for non-nucleated pearls than in those cultured for visceral mass-nucleated and mantle-nucleated pearls (Holm-adjusted *p* < 0.05), while their concentrations were similar between the latter two groups (Holm-adjusted *p* > 0.05). Zn was similar between mussels cultured for visceral mass-nucleated and mantle-nucleated pearls (Holm-adjusted *p* > 0.05), but significantly higher than in mussels cultured for non-nucleated pearls (Holm-adjusted *p* < 0.05). The remaining elements showed similar concentrations among the three groups (Holm-adjusted *p* > 0.05).

In the pearls, K, Fe, Co, Ni, Se, and Mo were significantly higher in mussels cultured for non-nucleated pearls than in those cultured for visceral mass-nucleated and mantle-nucleated pearls (Holm-adjusted *p* < 0.05), while their concentrations were similar between the latter two groups (Holm-adjusted *p* > 0.05). Mg and Sr were similar between mussels cultured for non-nucleated and visceral mass-nucleated pearls (Holm-adjusted *p* > 0.05), but significantly higher than in mussels cultured for mantle-nucleated pearls (Holm-adjusted *p* < 0.05). Mn and Ba differed significantly among all three groups (Holm-adjusted *p* < 0.05), with the highest concentrations in mussels cultured for non-nucleated pearls and the lowest in those cultured for mantle-nucleated pearls. Al differed significantly only between mussels cultured for non-nucleated and mantle-nucleated pearls (Holm-adjusted *p* < 0.05). Na was similar between mussels cultured for visceral mass-nucleated and mantle-nucleated pearls (Holm-adjusted *p* > 0.05), but significantly lower than in mussels cultured for non-nucleated pearls (Holm-adjusted *p* < 0.05).

#### 3.4.2. Principal Component Analysis

**Figure 8:**
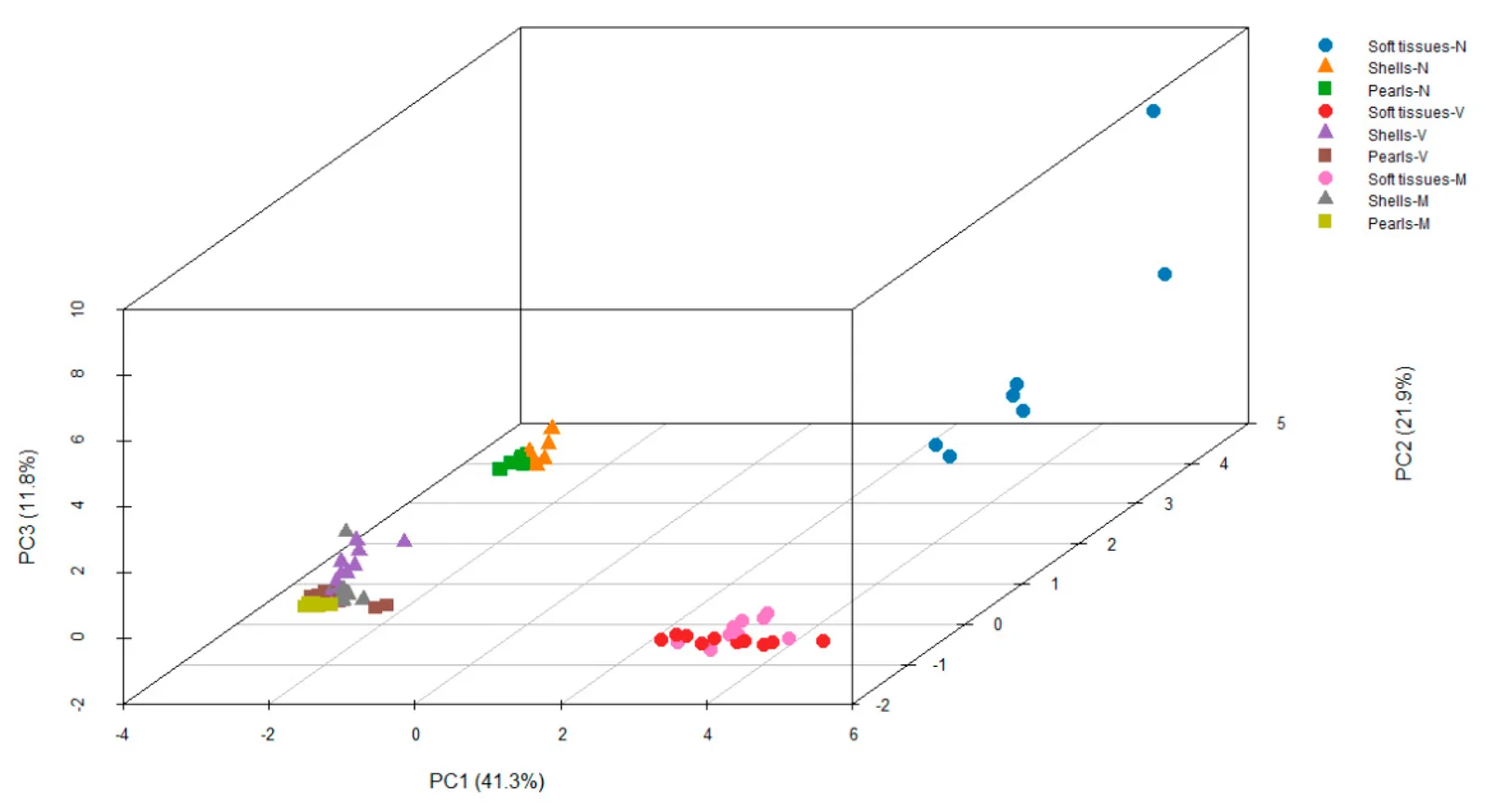
Principal component analysis of mineral elements in the soft tissues, shells, and pearls of *Sinohyriopsis cumingii* cultured for different types of pearls. Soft tissues-N, soft tissues of *S. cumingii* cultured for non-nucleated pearls (*n* = 7); Shells-N, shells of *S. cumingii* cultured for non-nucleated pearls (*n* = 7); Pearls-N, pearls of *S. cumingii* cultured for non-nucleated pearls (*n* = 7); Soft tissues-V, soft tissues of *S. cumingii* cultured for visceral mass-nucleated pearls (*n* = 10); Shells-V, shells of *S. cumingii* cultured for visceral mass-nucleated pearls (*n* = 10); Pearls-V, pearls of *S. cumingii* cultured for visceral mass-nucleated pearls (*n* = 10); Soft tissues-M, soft tissues of *S. cumingii* cultured for mantle-nucleated pearls (*n* = 10); Shells-M, shells of *S. cumingii* cultured for mantle-nucleated pearls (*n* = 10); Pearls-M, pearls of *S. cumingii* cultured for mantle-nucleated pearls (*n* = 10).

As shown in Figure 8, PC1, PC2, and PC3 cumulatively explained 75.0% of the variation in mineral element concentrations among the soft tissues, shells, and pearls of mussels cultured for different pearl types. The main contributing elements to PC1 were Zn (12.81%), Ca (12.70%), Mg (12.26%), Sr (11.54%), and Mn (11.37%), which distinguished soft tissues from shells and pearls. The main contributing elements to PC2 were Mo (24.11%), Fe (18.39%), Co (16.04%), and Ni (15.77%), which distinguished mussels cultured for non-nucleated pearls from those cultured for nucleated pearls. The main contributing elements to PC3 were Cu (25.34%), Cd (21.99%), and As (11.91%), which distinguished the soft tissues of mussels cultured for non-nucleated pearls.

#### 3.4.3. Correlation Analysis

Correlation analyses of mineral elements were performed within and between the soft tissues, shells, and pearls of mussels cultured for the three pearl types. Ca–Sr and Sr–Ba were consistently positively correlated in the soft tissues across all three pearl mussel types (FDR-adjusted *p* < 0.05), whereas no common correlated element pairs were found in the shells or pearls. Between tissues, no common correlated element pairs were shared across the three pearl mussel types. Nevertheless, Na appeared to act as a negative correlation hub. For example, in mussels cultured for non-nucleated pearls, Na in the shells was significantly negatively correlated with K in the soft tissues (FDR-adjusted *p* < 0.05), and Na in the pearls was significantly negatively correlated with Ba in the soft tissues (FDR-adjusted *p* < 0.05). In mussels cultured for visceral mass-nucleated pearls, Na in the soft tissues was significantly negatively correlated with both Mn and Zn in the pearls (FDR-adjusted *p* < 0.05). In mussels cultured for mantle-nucleated pearls, Na in the pearls was significantly negatively correlated with Zn in the shells (FDR-adjusted *p* < 0.05).

## 4. Discussion

### 4.1. Mineral Element Bioaccumulation in Soft Tissues, Shells, and Pearls

Freshwater pearl mussels typically accumulate mineral elements in their soft tissues through two primary pathways [10,22]: direct absorption of mineral elements from the aquatic environment through the gills and mantle, and uptake from food through the digestive system. Subsequently, these mineral elements are transported throughout the soft tissues via the haemolymph [10].

This study revealed that the soft tissues of *S. cumingii* cultured for non-nucleated pearls accumulated a significantly greater diversity of mineral elements than those of mussels cultured for nucleated pearls (visceral mass-nucleated and mantle-nucleated pearls). In addition, the concentrations of several mineral elements, including Ca, Cr, Mn, Fe, As, Sr, Mo, and Ba, were higher in mussels cultured for non-nucleated pearls. All mussels used in this study were cultured under the same environmental conditions and had similar body sizes. However, the three groups differed simultaneously in pearl-culture method, mussel age, and culture duration: mussels producing non-nucleated pearls were three years old with a two-year culture period, whereas those producing nucleated pearls were two years old with a one-year culture period. This difference reflects the fact that non-nucleated pearls require a longer cultivation period to reach commercial size owing to their slower nacre deposition. Nevertheless, because these factors are inseparable in the present design, the observed differences in mineral element accumulation among the three groups cannot be attributed to any single factor. The higher elemental accumulation observed in mussels producing non-nucleated pearls may be related to their longer culture duration, older age, or the non-nucleated culture method itself. Despite these differences, the soft tissues generally exhibited a concentration pattern of essential major elements (e.g., Ca, K, Na, and Mg) > essential trace elements (e.g., Fe and Zn) > non-essential elements (e.g., Sr, Al, and As), with the exception of Mn, which showed an unusually high concentration despite being classified as an essential trace element. Similar patterns of elevated Mn accumulation have also been reported in other freshwater bivalves, such as *Sinanodonta woodiana* (Lea, 1834), in which Mn concentrations in soft tissues ranged from 2933 to 3560 μg/g dry weight [18]. A previous study showed that freshwater mussels, such as *Hyridella depressa* (Lamarck, 1819) and *Velesunio ambiguous* (Philippi, 1847), absorb Mn as an analogue of Ca [23]. Additionally, high Mn accumulation in freshwater bivalves like *Unio pictorum mancus* (Linnaeus, 1758) can exert antioxidant effects [24]. Therefore, we propose that *S. cumingii* achieves high Mn accumulation through both active and passive pathways.

The shells of freshwater mussels are formed by biomineralization involving the secretion of biomacromolecules (e.g., matrix proteins) and ions (e.g., Ca^2+^, HCO^3−^, Na^+^, K^+^, Mg^2+^) by the mantle [10,25]. The shells are primarily composed of calcium carbonate (CaCO_3_), accounting for 95–96% of the shell’s dry weight [26]. Consequently, the Ca concentration in the shell of *S. cumingii* is much higher than that of other mineral elements, being more than 155 times higher than Na, the second-highest concentration. Even with longer culture times (3 years for mussels producing non-nucleated pearls vs. 2 years for mussels producing nucleated pearls), the Ca concentration in the shell did not change significantly. Nevertheless, increased culture time led to a greater variety of mineral elements accumulated in the shell.

The pearl is formed by the proliferation and differentiation of the small piece of mantle tissue into a pearl sac, followed by the secretion of various biomacromolecules and ions required for biomineralization by the epithelial cells of the pearl sac, ultimately forming the nacre that envelops the pearl nucleus [10]. The main component of nacre is also CaCO_3_ (approximately 95%) [10]. Therefore, the Ca concentration in all three pearl types was much higher than that of other mineral elements, being 144–157 times higher than Na, the second-highest concentration. Nevertheless, the variety and concentrations of many elements accumulated in non-nucleated pearls were significantly higher than those in nucleated pearls. For example, non-nucleated pearls had the highest concentrations of K, Fe, Co, Ni, Se, Mo, Mn, and Ba. This may be related to the longer culture period for non-nucleated pearls. It is worth noting that not all mineral elements with significant concentration differences were accumulated most in the non-nucleated pearls. For instance, visceral mass-nucleated pearls had the highest Zn concentration, while mantle-nucleated pearls had the highest Na concentration. This may be related to differences in biomineralization mechanisms between the mantle and visceral mass [27] of *S. cumingii*.

### 4.2. Principal Components of Element Accumulation in Soft Tissues, Shells, and Pearls

The PCA explained 75.0–85.0% of the variation in mineral element concentrations among the soft tissues, shells, and pearls of the three pearl mussel types in the present study. Among these, Zn, Ca, Mg, Sr, and Mn could distinguish the soft tissues from the hard tissues (including shells and pearls) of *S. cumingii*. Zn, Mg, and Mn are essential elements, serving as components and activators of many enzymes, such as alkaline phosphatase, phosphate transferase, and decarboxylases [28]. This study showed that Zn, Mg, and Mn tend to accumulate in the soft tissues of *S. cumingii*. This is similar to the tissue accumulation characteristics of Zn, Mg, and Mn in *S. woodiana* [18,21]. However, the CaCO_3_ content in both shells and pearls is around 95% [10,26], resulting in significantly higher Ca concentrations in these hard tissues than in soft tissues. Although Sr is a non-essential element, Sr^2+^ has a similar ionic radius and the same charge as Ca^2+^, allowing Sr^2+^ to substitute for Ca^2+^ in the calcium carbonate lattice via isomorphism during biomineralization [29], consequently leading to significantly higher Sr concentrations in the hard tissues of *S. cumingii* compared to soft tissues. This indicates clear differences in the metabolism and storage functions of Zn, Ca, Mg, Sr, and Mn between the soft and hard tissues of *S. cumingii*. Furthermore, Mo, Fe, Co, and Ni concentrations were significantly higher in non-nucleated pearl mussels (across soft tissues, shells, and pearls) than in nucleated ones, effectively separating the two groups. Although Mo, Co, and Ni concentrations in the water are very low [15], prolonged culture still resulted in higher accumulation of Mo, Fe, Co, and Ni in both soft and hard tissues of *S. cumingii*.

It is worth noting that the 21 mineral elements studied could not distinguish between the shells and pearls of non-nucleated and nucleated pearl mussels. This indicates that the mineral element composition of shells and pearls is very similar. This may be related to the similar formation mechanisms of shells and pearls, both being formed by biomineralization involving the secretion of biomacromolecules and ions by mantle epithelial cells [10,25]. Similarly, shells and pearls of *Hyriopsis schlegeli* (Martens, 1861) also share very similar mineral element compositions [30].

### 4.3. Correlations of Element Accumulation in Soft Tissues, Shells, and Pearls

Correlation analysis revealed multi-level associations of mineral element bioaccumulation within *S. cumingii* cultured for three pearl types. Despite certain differences in culture methods and duration, consistent patterns emerged in the elemental correlations within and between tissues. This suggests that targeted regulation of the bioaccumulation of certain mineral elements in *S. cumingii* is potentially feasible.

In the soft tissues of mussels cultured for the three pearl types, elemental correlations were mainly centered around Ca, Sr, and Ba. Similarly, in the soft tissues of *S. woodiana*, Sr was positively correlated with Ca and Ba [31], and in the soft tissues of *Lamprotula leai* (Gray, 1834), Sr-Ca was positively correlated [31]. This reflects the synergistic absorption and transport of these divalent cations in freshwater mussels. Apart from these, no common elemental correlations were found within or between other tissues among the three pearl culture types. Interestingly, however, Na appeared to act as a negative correlation hub, suggesting that Na may play a role in the accumulation of K, Ba, Mn, and/or Zn in the soft tissues, shells, and/or pearls of pearl mussels. Mg and Zn are key factors influencing pearl color [11,12,13]. Shi et al. [11] revealed that in *S. cumingii*, Mn concentration was highest in white pearls and Zn concentration was highest in purple pearls, while orange pearls showed intermediate levels of both. Therefore, in mussels cultured for visceral mass-nucleated pearls, the negative correlations between soft tissue Na and pearl Mn and Zn suggest a hypothesis that should be tested in future controlled experiments to determine whether manipulating Na or related elements can influence pearl color.

## 5. Conclusions

This study comprehensively compared the bioaccumulation characteristics of 21 mineral elements in the soft tissues, shells, and pearls of *S. cumingii* cultured for non-nucleated pearls, visceral mass-nucleated pearls, and mantle-nucleated pearls. Tissue-specific accumulation patterns were observed: Mg, K, Mn, Zn, and Ba preferentially accumulated in soft tissues, whereas Ca and Sr were predominantly enriched in shells and pearls. The PCA based on mineral element profiles effectively distinguished mussels cultured for non-nucleated pearls from those cultured for nucleated pearls, and separated soft tissues from hard tissues, while it could not clearly differentiate shells from pearls. Significant correlations among mineral elements were identified both within and between tissues. In particular, Ca–Sr and Sr–Ba were consistently positively correlated in the soft tissues across all three pearl mussel types, and Na appeared to act as a negative correlation hub between tissues. However, pearl-culture method, mussel age, and culture duration differed simultaneously in the present design. Their relative contributions therefore remain undetermined, and future age- and duration-matched studies are needed. Moreover, since environmental element concentrations were not experimentally manipulated and pearl-color responses were not tested, the implications of these findings for targeted elemental regulation or pearl-quality management should be regarded as hypotheses for future controlled studies rather than demonstrated applications.

## Figures and Tables

**Table 1:** Mineral element concentrations in soft tissues, shells, and pearls from *Sinohyriopsis cumingii* cultured for non-nucleated pearls (μg/g dry weight, mean ± SD, *n* = 7).

Mineral Elements	Soft Tissues	Shells	Pearls
Na	1694 ± 706 ^a^	20,006 ± 159 ^a^	2032 ± 49.67 ^a^
Mg	1085 ± 221 ^a^	159 ± 5.83 ^b^	156 ± 9.80 ^b^
K	829 ± 186 ^a^	208 ± 6.71 ^b^	209 ± 5.95 ^b^
Ca	22,331 ± 5840 ^b^	309,513 ± 27,163 ^ab^	319,510 ± 3806 ^a^
Cr	6.72 ± 1.91 ^a^	4.71 ± 0.74 ^ab^	2.26 ± 0.05 ^b^
Mn	4072 ± 943 ^a^	499 ± 77.53 ^b^	666 ± 134 ^ab^
Fe	2270 ± 540 ^b^	14,243 ± 1307 ^a^	14,034 ± 209 ^a^
Co	0.04 ± 0.10 ^b^	2.40 ± 0.35 ^a^	2.29 ± 0.07 ^a^
Ni	0.11 ± 0.30 ^b^	5.07 ± 0.85 ^a^	3.65 ± 0.20 ^ab^
Cu	0.23 ± 0.40 ^a^	nd ^a^	nd ^a^
Zn	200 ± 29.41 ^a^	nd ^b^	nd ^b^
Se	nd ^a^	nd ^a^	0.30 ± 0.47 ^a^
Al	19.70 ± 7.96 ^a^	10.99 ± 2.13 ^a^	3.09 ± 1.33 ^b^
As	5.69 ± 1.98 ^a^	nd ^b^	nd ^b^
Sr	178 ± 41.15 ^b^	630 ± 39.71 ^ab^	799 ± 42.46 ^a^
Mo	1.97 ± 0.09 ^a^	1.86 ± 0.10 ^ab^	1.63 ± 0.04 ^b^
Cd	0.03 ± 0.08 ^a^	nd ^a^	nd ^a^
Ba	786 ± 223 ^a^	164 ± 15.92 ^b^	274 ± 58.89 ^ab^

nd, not detected; Ag, Tl, and Pb were excluded as they were not detected in any samples; Different superscript letters within the same row denote significant differences at *p* < 0.05.

**Table 2:** Mineral element concentrations in soft tissues, shells, and pearls from *Sinohyriopsis cumingii* cultured for visceral mass-nucleated pearls (μg/g dry weight, mean ± SD, *n* = 10).

Mineral Elements	Soft Tissues	Shells	Pearls
Na	1275 ± 307 ^b^	1924 ± 84.51 ^a^	1947 ± 178 ^a^
Mg	1048 ± 125 ^a^	112 ± 5.49 ^b^	201 ± 49.01 ^ab^
K	1785 ± 193 ^a^	nd ^b^	nd ^b^
Ca	11,910 ± 1961 ^b^	324,858 ± 7340 ^a^	319,659 ± 13,896 ^a^
Cr	nd ^b^	7.07 ± 4.59 ^a^	nd ^b^
Mn	2269 ± 365 ^a^	466 ± 48.84 ^b^	358 ± 112 ^b^
Fe	292 ± 92.10 ^a^	nd ^b^	nd ^b^
Zn	163 ± 20.81 ^a^	9.58 ± 2.28 ^b^	13.02 ± 3.94 ^b^
Al	33.69 ± 29.58 ^a^	5.12 ± 11.27 ^b^	4.79 ± 8.33 ^ab^
As	0.18 ± 0.30 ^a^	nd ^a^	nd ^a^
Sr	86.83 ± 12.27 ^b^	703 ± 42.25 ^a^	733 ± 163 ^a^
Ba	226 ± 41.36 ^a^	107 ± 8.75 ^b^	134 ± 26.50 ^b^

nd, not detected; Co, Ni, Cu, Se, Mo, Ag, Cd, Tl, and Pb were excluded as they were not detected in any samples; Different superscript letters within the same row denote significant differences at *p* < 0.05.

**Table 3:** Mineral element concentrations in soft tissues, shells, and pearls from *Sinohyriopsis cumingii* cultured for mantle-nucleated pearls (μg/g dry weight, mean ± SD, *n* = 10).

Mineral Elements	Soft Tissues	Shells	Pearls
Na	1552 ± 219 ^b^	1833 ± 137 ^b^	2232 ± 99.27 ^a^
Mg	1176 ± 112 ^a^	110 ± 7.25 ^b^	113 ± 12.75 ^b^
K	1904 ± 144 ^a^	nd ^b^	nd ^b^
Ca	13,863 ± 2201 ^b^	324,375 ± 21,554 ^a^	321,027 ± 10,160 ^a^
Cr	nd ^b^	2.39 ± 4.32 ^a^	nd ^b^
Mn	2880 ± 584 ^a^	515 ± 87.96 ^ab^	213 ± 74.33 ^b^
Fe	306 ± 57.65 ^a^	nd ^b^	nd ^b^
Zn	188 ± 24.88 ^a^	13.03 ± 11.21 ^b^	5.23 ± 6.28 ^b^
Se	nd ^a^	0.03 ± 0.07 ^a^	nd ^a^
Al	15.57 ± 11.51 ^a^	2.04 ± 0.72 ^b^	1.32 ± 0.48 ^b^
As	0.54 ± 0.80 ^a^	nd ^a^	nd ^a^
Sr	99.14 ± 11.52 ^b^	681 ± 78.57 ^a^	552 ± 44.32 ^a^
Ba	250 ± 37.36 ^a^	106 ± 12.03 ^b^	74.43 ± 8.94 ^b^

nd, not detected; Co, Ni, Cu, Mo, Ag, Cd, Tl, and Pb were excluded as they were not detected in any samples; Different superscript letters within the same row denote significant differences at *p* < 0.05.

## Data Availability

The original contributions presented in this study are included in the article. Further inquiries can be directed to the corresponding author.
